# Functional decoupling of atrazine biodegradation and microbial respiration under viral modulation

**DOI:** 10.1128/spectrum.01812-26

**Published:** 2026-09-17

**Authors:** Yongfeng Wang, Lu Yang, Xiaolong Liang

**Affiliations:** 1 CAS Key Laboratory of Forest Ecology and Silviculture, Institute of Applied Ecology, Chinese Academy of Sciences 74763, Shenyang, China; 2 The Benioff Center for Microbiome Medicine, University of California San Francisco 8785, San Francisco, California, USA; Ruhr-Universitat Bochum, Bochum, Germany

**Keywords:** atrazine degradation, toxic stress, microbial respiration, soil microcosm, viral mediation

## Abstract

Atrazine persistence in agricultural soils is primarily controlled by microbial degradation. However, the combined roles of contaminant dose and viral communities in shaping microbial community assembly and biodegradation potential remain critically underexplored. Here, we established a simplified sand microcosm inoculated with a soil-derived microbial consortium to investigate how atrazine dose and viral communities jointly influence atrazine dissipation, bacterial community assembly, and microbial respiration. Atrazine degradation exhibited a clear dose-dependent response. Atrazine in the low-dose treatment (20 mg/kg) was completely degraded within 33 days, whereas most of it remained in the high-dose treatment (100 mg/kg), indicating a severe suppression of biodegradation capacity under elevated toxic stress. This divergence corresponded to fundamentally distinct assembly trajectories. Low-dose atrazine rapidly enriched Pseudomonadota-dominated communities, whereas high-dose atrazine initially selected Bacillota-dominated stress-tolerant assemblages. Alpha diversity declined sharply following atrazine exposure, with microbial richness exhibiting stronger dose sensitivity than Shannon diversity. Beta nearest taxon index analysis further revealed strong early homogeneous selection, followed by progressively weaker deterministic or stochastic assembly over time. Despite these strong dose-dependent effects on community structure and biodegradation, viral addition did not significantly alter atrazine degradation kinetics or most bacterial compositional metrics. In contrast, viruses significantly suppressed cumulative microbial respiration under the low-dose regime, demonstrating that viral regulation can alter community-scale carbon metabolism without measurably affecting specialized biodegradation capacity. Collectively, these findings reveal a functional decoupling between atrazine biodegradation and microbial respiration under viral modulation, highlighting that distinct microbial ecosystem functions differ fundamentally in their sensitivity to contaminant stress and viral regulation.

**IMPORTANCE:**

Predicting contaminant effects on soil microbiomes generally relies on the assumption that microbial functions respond coherently to environmental disturbance. By integrating community assembly analysis and viral manipulation experiments across atrazine dosages, this study demonstrates that contaminant concentration acts as a master variable governing microbial community trajectory. Increasing atrazine dosage shifts assembly from degrader-dominated states to stress-persistent communities. At the functional level, specialized biodegradation and community-scale metabolism exhibit fundamentally different sensitivities. Atrazine degradation remains resilient to viral perturbation, while respiration is strongly suppressed. Viral regulation of carbon flux operates independently of contaminant removal. These findings identify contaminant concentration as a key ecological threshold that decouples community assembly and functional specialization.

## INTRODUCTION

Atrazine (2-chloro-4-ethylamino-6-isopropylamino-1,3,5-triazine) is one of the most extensively used triazine herbicides in global agricultural production, particularly in maize, sorghum, and sugarcane systems (1). Despite its agricultural benefits, atrazine has raised substantial ecological and human health concerns (2). Its environmental persistence and high mobility have led to long-term accumulation in agricultural soils and frequent detection in groundwater and surface waters (3, 4). Consequently, atrazine has become a model contaminant for understanding how chronic chemical stress reshapes soil ecosystems. Although atrazine was banned for future use in the European Union (5), it continues to be heavily applied in several major agricultural regions worldwide. Understanding how soil microbial systems respond to atrazine exposure is therefore essential not only for predicting contaminant persistence but also for evaluating broader impacts on soil ecological functioning.

Microbial degradation is the dominant mechanism controlling atrazine removal from soils. Diverse bacterial taxa possess catabolic pathways capable of transforming atrazine and its intermediates, enabling atrazine-derived carbon and nitrogen to enter microbial metabolism (6). However, biodegradation potential is not determined solely by the presence of degrader taxa. Instead, degradation emerges from the interaction between contaminant stress, microbial physiology, and ecological selection (7). Consequently, contaminant dose may represent a critical ecological threshold determining whether microbial communities transition toward functional biodegradation or toward stress persistence with limited catabolic capacity.

Recent studies increasingly demonstrate that pesticides can alter soil microbiomes far beyond their intended target effects, reshaping microbial diversity, ecological interactions, and biogeochemical functioning (8, 9). Atrazine exposure has been linked to reductions in microbial biomass, shifts in dominant bacterial taxa, and suppression of soil enzyme activities (10, 11). However, these studies have primarily described compositional responses without explicitly connecting contaminant dose, community assembly processes, and ecosystem functional outcomes within a unified ecological framework. Understanding contaminant responses therefore requires moving beyond purely compositional descriptions toward an integrated analysis linking degradation kinetics, microbial assembly, and ecosystem metabolic activity.

The atrazine fate in agricultural soils is governed by microbial degradation, as specialized bacterial communities have evolved catabolic capacities enabling them to utilize atrazine as a carbon and nitrogen source. However, this degradation potential can be impacted by the concentration of atrazine, which can inhibit even specialized degrader populations, creating a threshold response beyond which catabolic activity ceases. Moreover, atrazine residues may modulate ecosystem-level outcomes by altering microbiome composition, with potential consequences for nutrient cycling and plant–soil interactions. Collectively, these findings underscore the need to understand how chemical dose shapes microbial community assembly and functional outcomes.

Soil viruses introduce an additional and largely unresolved dimension to contaminant-driven microbial ecology. Viruses are increasingly recognized as important regulators of microbial population dynamics, metabolic activity, and biogeochemical cycling in soils (12, 13). Through lytic infection, viruses can suppress dominant microbial populations and alter carbon fluxes, whereas lysogenic interactions and virus-encoded auxiliary metabolic genes may reprogram host metabolism under environmental stress (14). Viral genes associated with xenobiotic transformation and pesticide metabolism have been identified in contaminated environments, suggesting a potential role for viruses in regulating contaminant-processing pathways (15, 16). Moreover, recent work demonstrated that viral communities could influence atrazine biodegradation under different nutrient conditions (17). Yet, despite growing recognition of viral ecological importance, it remains unclear whether viral effects under contaminant stress primarily influence biodegradation capacity, microbial community assembly, or broader metabolic functions such as respiration.

A central unresolved question is therefore whether different microbial ecosystem functions exhibit similar or distinct sensitivities to contaminant stress and viral regulation. To address this question, we established a reduced-complexity sand microcosm inoculated with soil-derived bacterial and viral fractions to isolate the ecological effects of atrazine dose and viral addition from the strong physicochemical heterogeneity of natural soils. Specifically, we aimed to determine (i) whether atrazine dose establishes a threshold between active biodegradation and toxic inhibition; (ii) how dose-dependent stress shapes bacterial diversity, taxonomic succession, and community assembly dynamics; and (iii) whether viral addition differentially regulates atrazine degradation, bacterial community composition, and microbial respiration. We hypothesized that low atrazine dose would enrich specialized degrader-dominated communities and sustain biodegradation, whereas high dose would impose strong toxic filtering that suppresses catabolic activity and favors stress-tolerant taxa. We further hypothesized that viral effects would be expressed more strongly at the level of microbial metabolic activity than taxonomic structure, potentially generating a functional decoupling between atrazine biodegradation and respiratory carbon loss under contaminant stress.

## MATERIALS AND METHODS

### Soil sample collection

Soil samples were collected from a depth of 0–20 cm in an agricultural field in July 2023. Immediately after collection, the samples were transported to the laboratory on dry ice to preserve field conditions. Upon arrival, each sample was passed through a 2 mm sieve to remove coarse debris. Visible plant residues (e.g., roots, leaves, and stubble) and stones were removed. The soil was then stored at –20°C prior to extraction.

### Soil virus and bacterial extraction

Soil viruses and bacteria were extracted following our previous protocol with minor modifications (17). Briefly, soil was suspended in potassium citrate buffer solution (10 g C_6_H_5_K_3_O_7_, 1.44 g Na_2_HPO_4_⋅7H_2_O, and 0.24 g KH_2_PO_4_ per liter; pH adjusted to 7; stored at 4°C) at a 3:1 liquid-to-soil ratio (vol/wt). The suspension was transferred into an autoclaved blender vessel and blended at maximum speed for 3 min. The resulting slurry was transferred into 50 mL polypropylene conical tubes (Labselect, China) and centrifuged at 5,000 × *g* for 20 min at 4°C. The supernatant was filtered through a bottle-top vacuum filter with a 0.22 μm polyethersulfone membrane (Corning, USA). The filtrate was retained as the virus-containing filtrate. The sediments on the filter were washed three times and then resuspended in 100 mL of phosphate-buffered saline buffer, which served as the bacterial extraction solution.

### Incubation experiment

The incubation experiment was conducted in 500 mL serum flasks containing 300 g of sterilized sand (dry weight). Sterilized sand was selected as the microcosm substrate to provide a minimal and reproducible matrix with controlled physicochemical properties. Sand eliminates the confounding effects of soil organic matter, clay mineralogy, and native microbial communities. The sand was spiked with 600 μL of an atrazine stock solution to achieve the desired initial concentration. After complete evaporation of the solvent, the spiked sand was thoroughly mixed with 20 mL of virus extract, 20 mL of bacterial extract, and 25 mL of minimal nutrient medium. To assess abiotic atrazine transformation, control flasks were prepared using sterilized biological extracts. Sterilization was achieved by autoclaving at 121°C for 30 min. The experiment totally comprised 18 jars (2 atrazine doses × 3 inoculum treatments × 3 biological replicates). Soils were incubated at 25°C in the dark throughout the experimental period. The different samples were taken for analysis at different time points. Specifically, 50 mL of headspace gas was collected for CO_2_ analysis (days 0.5, 1, 2, 4.5, 7, 10, 15, 23, and 33), 5 g of sand for atrazine concentration analysis (days 0.5, 2, 7, 15, 23, and 33), and 5 g of sand for microbial community analysis (days 2, 15, and 33).

### Atrazine concentration analysis

To quantify atrazine, collected sand samples were extracted using methanol at a solid-to-liquid ratio of 1:3 (wt/vol) and shaken for 20 min. The mixture was then centrifuged at 6,000 × *g* for 20 min at 4°C. The supernatant was subsequently passed through 0.45 μm Millex syringe filters (Merck Millipore, Ireland). Atrazine concentrations in the filtrate were determined using a Thermo UltiMate 3000 high-performance liquid chromatography system (Thermo Fisher Scientific, Waltham, MA, USA) equipped with a diode array detector (DAD). A 20-μL aliquot of each sample was injected and separated on a Kinetex C18 column (100 × 4.6 mm, 2.6 μm particle size; Phenomenex, Torrance, CA, USA) maintained at ambient temperature. Isocratic elution was performed with a mobile phase consisting of 70% methanol and 30% water (vol/vol) at a flow rate of 1.0 mL/min. Atrazine and its metabolites were monitored at a wavelength of 240 nm.

### Gas sampling and analysis

Gas samples were analyzed within 48 h of collection using gas chromatography. A 50 mL headspace gas sample was withdrawn and analyzed with an Agilent 7890A gas chromatograph (Agilent Technologies, USA) equipped with a flame ionization detector (FID) for CO_2_ quantification. The CO_2_ evolution was used to quantify microbial respiration.

### DNA extraction, amplification, and sequencing

DNA was extracted from soil samples using the PowerLyser PowerSoil DNA Isolation Kit (Qiagen, Germany) following the manufacturer’s protocol. The V3–V4 region of the 16S rRNA gene was amplified using primers 338F and 806R. PCR conditions followed initial denaturation at 94°C for 3 min; 30 cycles of 94°C for 30 s, 52°C for 1 min, and 72°C for 30 s; and a final extension at 72°C for 10 min. PCR products were verified by 1% agarose gel electrophoresis, pooled, and purified using the E.Z.N.A. Gel Extraction Kit (Omega, USA). Libraries were prepared with the NEBNext Ultra II DNA Library Prep Kit (New England Biolabs, MA, USA), and quality was assessed using a Qubit 4.0 Fluorometer (Thermo Fisher Scientific, MA, USA). Sequencing was performed on an Illumina NovaSeq 6000 platform, generating 250 bp paired-end reads (Guangdong Magigene Biotechnology Co., Ltd., Guangzhou, China).

### Analysis of microbial community

Sequence data analysis was performed using Quantitative Insights Into Microbial Ecology (QIIME 2) following the platform’s tutorials (18). Raw sequence files containing forward and reverse reads were combined and imported as a QIIME 2 artifact. Quality control and denoising were performed using DADA2 (19), which generated amplicon sequence variants. Taxonomic assignment was performed using a pre-trained Naïve Bayes classifier (silva-138-99-515-806-nb-classifier) based on SILVA reference sequences (SILVA 16S v138, 99% similarity).

### Statistical analysis

All sequencing data analyses were performed using RStudio with the microeco package (20). Alpha diversity indices, including the Shannon diversity index and the Chao1 richness index, were calculated for each sample. Beta diversity was assessed using principal coordinate analysis (PCoA) based on Bray–Curtis distances. The beta nearest taxon index (βNTI) was calculated to infer the ecological processes driving microbial community assembly. A phylogenetic tree was constructed from 16S rRNA sequences using FastTree. For each pair of samples, the observed mean nearest taxon distance was computed and compared to a null distribution generated from 500 randomizations of the community matrix using the independent swap algorithm. Following established thresholds, |βNTI| >2 indicates deterministic selection (heterogeneous when >+2, homogeneous when <−2), while |βNTI| ≤2 indicates stochastic assembly. Atrazine degradation curves in different soils were plotted using Origin 2017 software. One-way analysis of variance (ANOVA) was performed using SPSS 26.0. Tukey’s honestly significant difference post hoc test was applied following ANOVA (*P* < 0.05). All analyses were performed on data from three independent biological replicates. Results are presented as mean ± standard deviation.

## RESULTS

### Atrazine dose determines degradation dynamics

**Fig 1:**
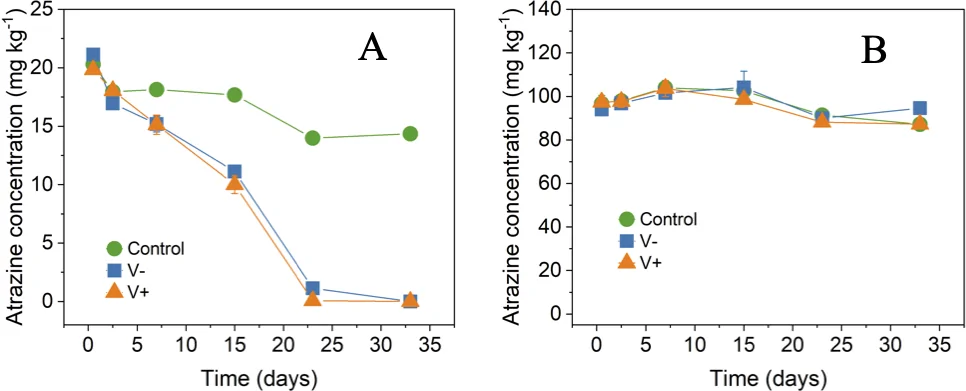
Atrazine degradation kinetics at low (**A**) and high (**B**) doses over 33 days. Control denotes sterilized treatment. V− and V+ denote virus-free and virus-present treatments.

Atrazine dissipation exhibited a pronounced dose-dependent threshold response (Fig. 1). Under the low-dose treatment (20 mg/kg), atrazine concentrations declined rapidly throughout the incubation and fell below the detection limit within 33 days (Fig. 1A), indicating efficient microbial biodegradation. In contrast, atrazine degradation was strongly suppressed under the high-dose treatment (100 mg/kg) (Fig. 1B), where most of the initially added atrazine persisted throughout the experiment. This divergence demonstrates that increasing contaminant loading fundamentally altered the functional capacity of the microbial system to sustain atrazine catabolism.

Importantly, degradation kinetics did not differ significantly between virus-present and virus-free treatments at either dosage level, indicating that viral addition exerted minimal influence on atrazine dissipation itself. In sterilized controls, approximately 88% of the added atrazine remained after 33 days, confirming that abiotic loss contributed minimally to atrazine removal and that degradation was overwhelmingly biologically mediated.

### Dose-dependent microbial community assembly under toxic stress

#### Alpha diversity responses

**Fig 2:**
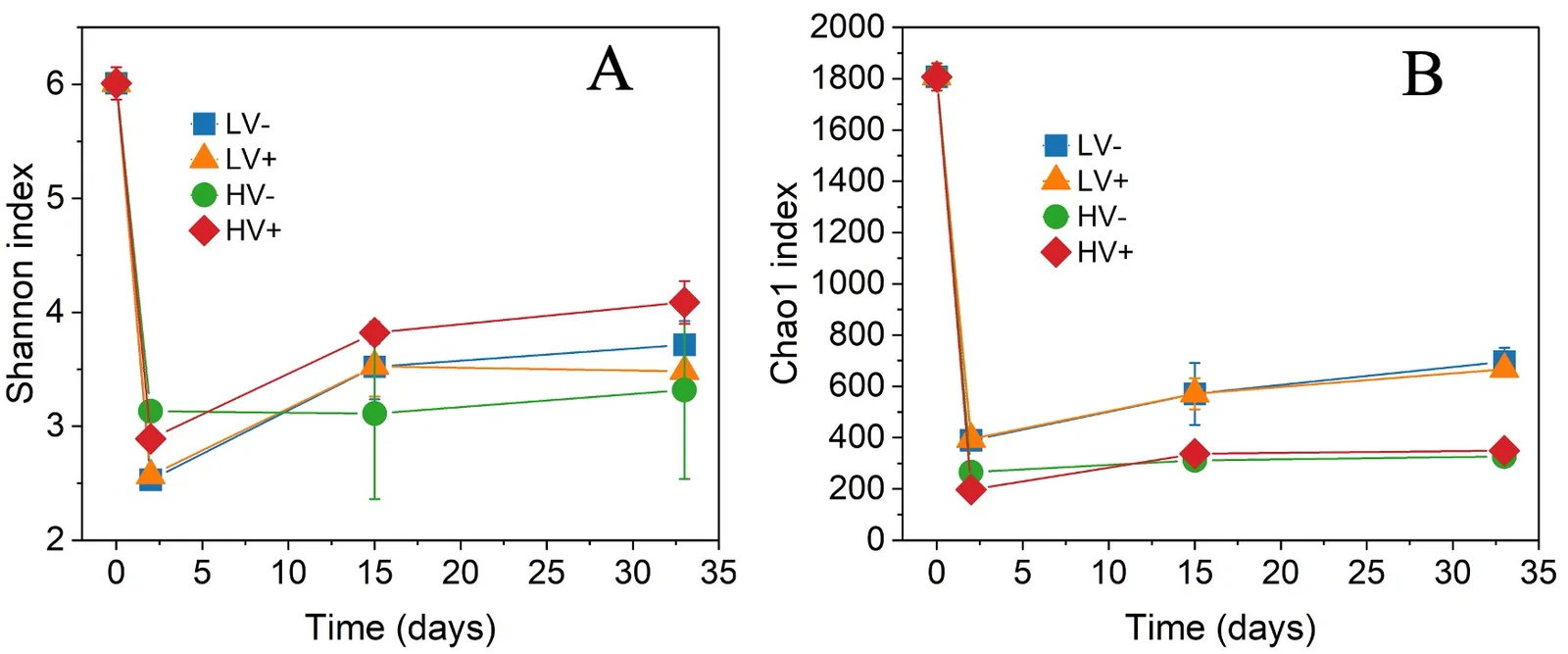
Alpha diversity of bacterial communities under different atrazine doses, shown as (**A**) Shannon index and (**B**) Chao1 index over time. LV−, LV+, HV−, and HV+ denote virus-free (V−) and virus-present (V+) treatments at low (L) and high (H) doses.

Atrazine exposure caused an immediate and substantial reduction in bacterial diversity across all treatments (Fig. 2). By day 2, Shannon diversity declined from 6.01 ± 0.14 in the initial inoculum to 2.78 ± 0.29, while Chao1 richness decreased from 1,806.83 ± 53.19 to 311.50 ± 97.16, indicating strong early ecological filtering following contaminant exposure.

The magnitude of diversity loss was dose dependent, although the response differed between diversity metrics. Chao1 richness exhibited consistently stronger suppression under the high-dose treatment throughout the incubation period (*P* < 0.05), indicating preferential loss of low-abundance taxa under elevated toxic stress. In contrast, Shannon diversity showed weaker long-term dose sensitivity, suggesting that atrazine primarily reduced richness rather than community evenness. Across all sampling points, viral addition did not significantly alter either Shannon or Chao1 diversity indices (*P* > 0.05 for all comparisons). These results demonstrate that atrazine toxicity rapidly simplified microbial communities and that richness was more sensitive to toxic stress than broader diversity structure.

#### Dose-dependent compositional divergence

**Fig 3:**
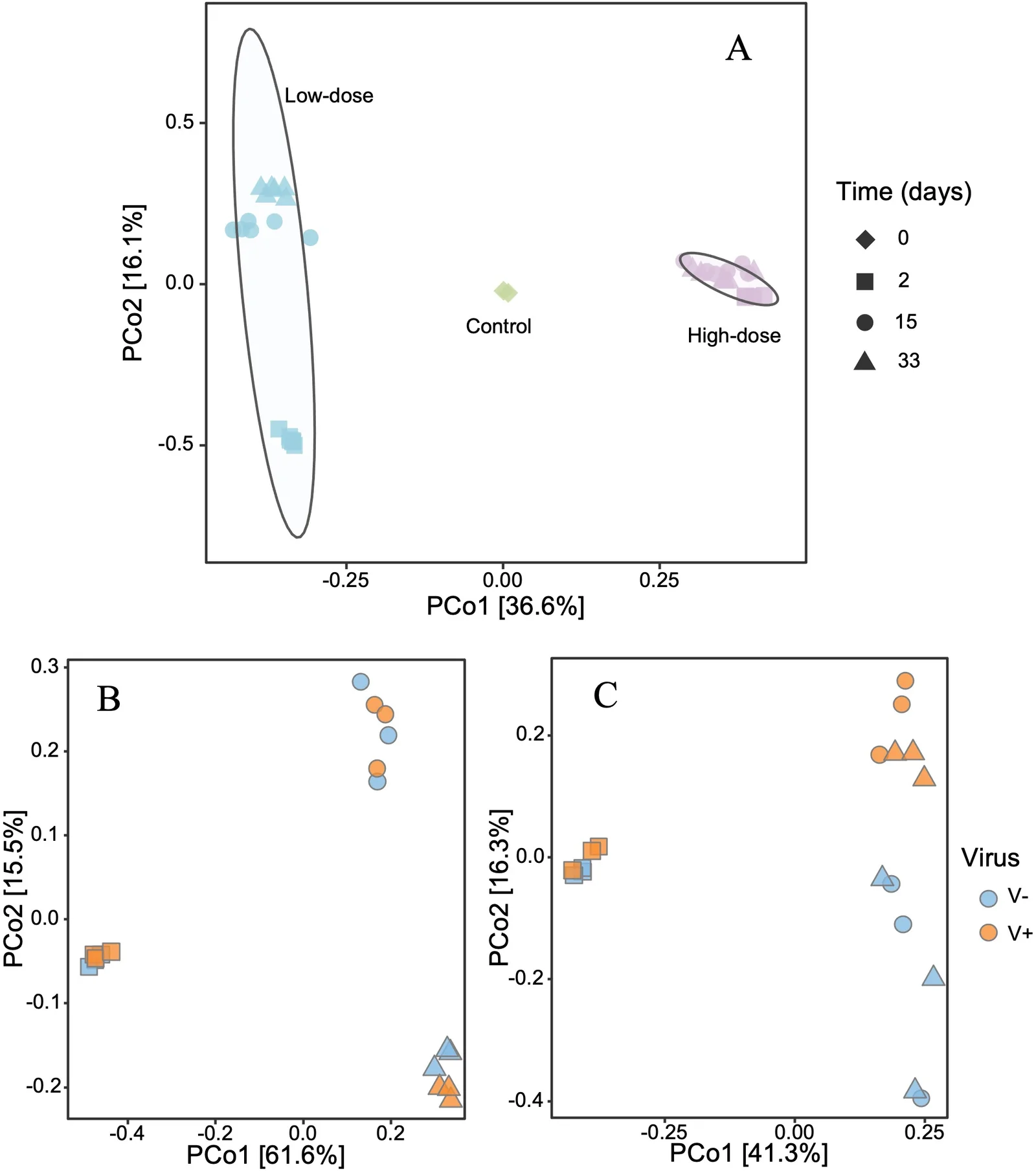
Principal coordinate analysis of bacterial communities under different atrazine doses (**A**), low dose (**B**), and high dose (**C**).

PCoA based on Bray–Curtis dissimilarity revealed strong compositional divergence between low- and high-dose treatments (Fig. 3A). Atrazine dosage represented the dominant factor structuring bacterial community composition, explaining 36.6% of the total variation along PCo1. Samples clustered distinctly according to contaminant dose, indicating that atrazine loading imposed fundamentally different ecological selection pressures on community assembly.

Within the low-dose treatment, virus-present and virus-free communities largely overlapped throughout the incubation (Fig. 3B), indicating minimal viral influence on overall taxonomic composition under conditions permitting active biodegradation. In contrast, partial separation between virus-present and virus-free communities emerged in the high-dose treatment at days 15 and 33 (Fig. 3C), suggesting that viral effects on community structure became detectable only under prolonged high-stress conditions.

**Fig 4:**
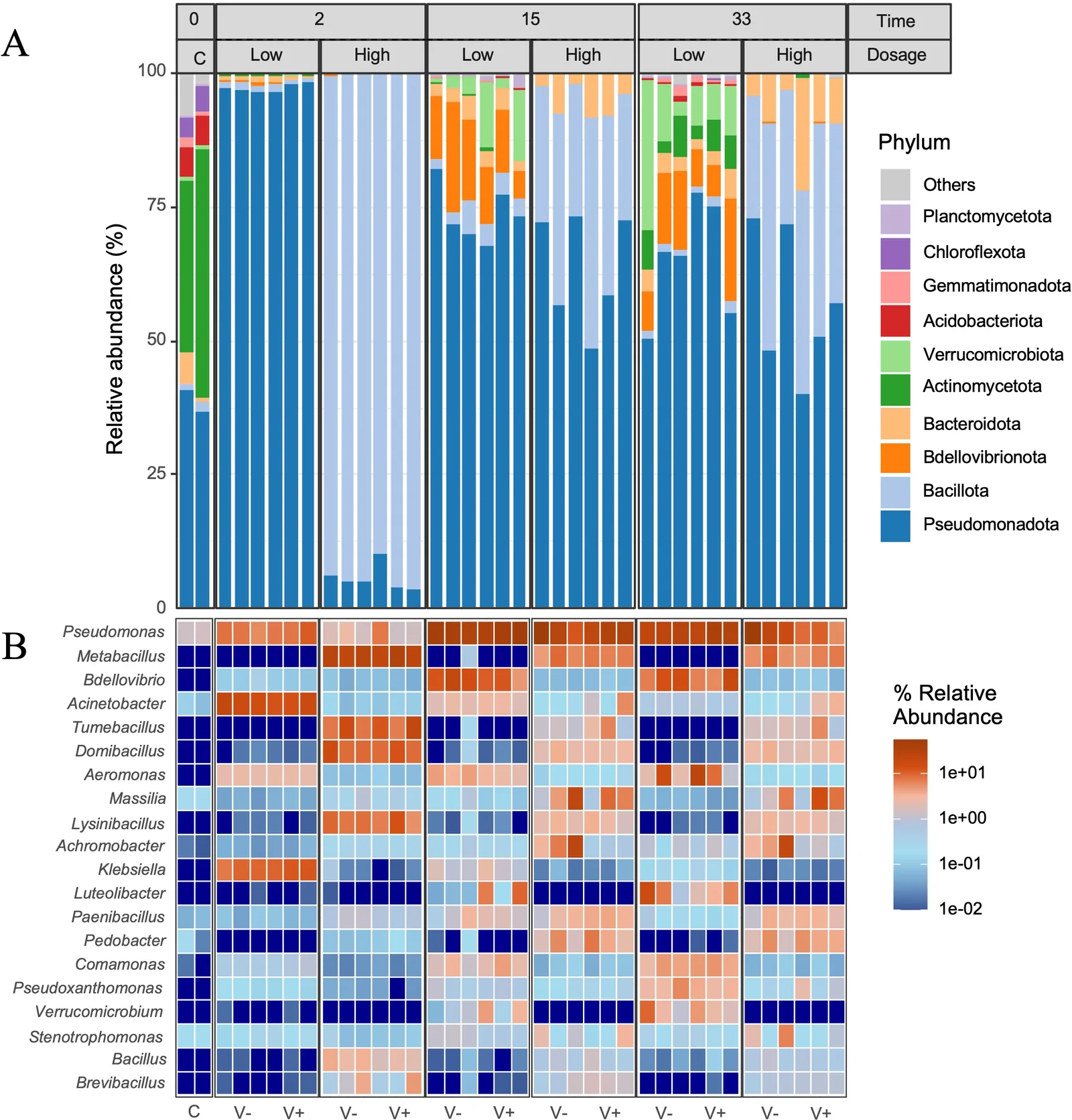
Microbial community composition under different atrazine doses at (**A**) the phylum level and (**B**) the genus level. C denotes sterilized treatment. V− and V + denote virus-free and virus-present treatments.

Taxonomic succession further demonstrated strong dose-dependent assembly trajectories (Fig. 4A). The initial inoculum was dominated primarily by Pseudomonadota and Actinobacteriota. Under the low-dose regime, Pseudomonadota rapidly became overwhelmingly dominant (>90% relative abundance), accompanied by enrichment of genera, including *Pseudomonas*, *Acinetobacter*, *Aeromonas*, and *Klebsiella* (Fig. 4B). In contrast, the high-dose treatment initially selected for Bacillota-dominated assemblages enriched in stress-tolerant genera such as *Pseudomonas*, *Metabacillus*, *Tumebacillus*, *Domibacillus*, *Lysinibacillus*, *Bacillus*, and *Brevibacillus* (Fig. 4B). Although partial recovery of Pseudomonadota occurred at later stages, the early dominance of Bacillota indicated strong toxic filtering toward stress-survival strategies rather than active catabolic specialization. Together, these results demonstrate that atrazine dose fundamentally redirected microbial assembly trajectories, selecting either degrader-dominated communities capable of sustaining biodegradation or stress-tolerant assemblages associated with functional suppression.

#### Community assembly dynamics under atrazine stress

**Fig 5:**
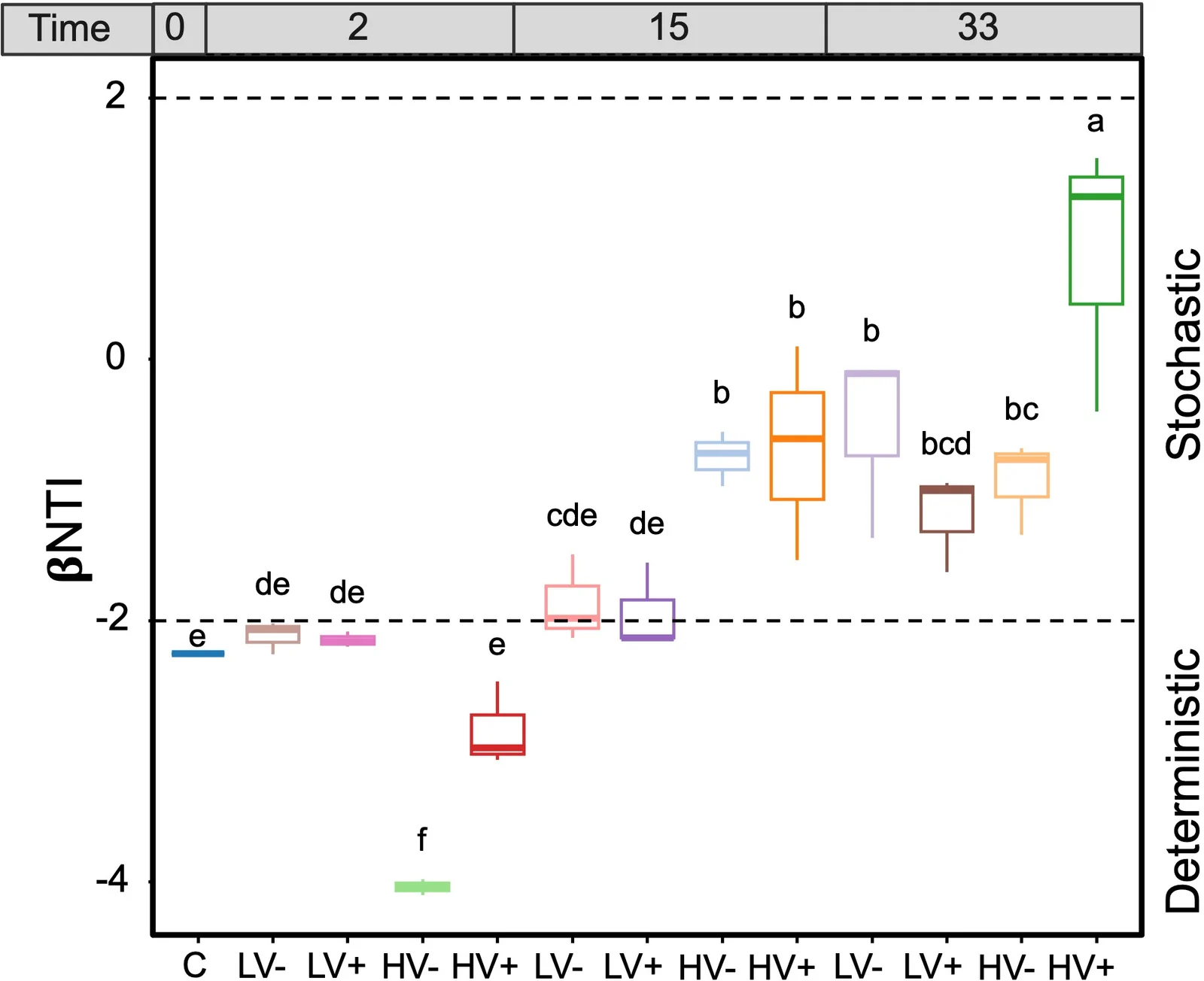
Microbial community assembly processes under different atrazine doses, inferred from βNTI values over time. C denotes sterilized treatment. LV−, LV+, HV−, and HV+ denote virus-free (V−) and virus-present (V+) treatments at low (L) and high (H) doses. The lowercase letters denote statistically significant differences among treatments (ANOVA with Tukey’s HSD, *P* < 0.05).

βNTI analysis revealed temporally dynamic and dosage-dependent assembly processes (Fig. 5). During the initial phase of incubation (days 0–2), βNTI values across treatments were consistently below −2, indicating strong homogeneous selection imposed by acute atrazine stress. This early deterministic filtering coincided with the rapid collapse in diversity and the pronounced taxonomic restructuring observed immediately following exposure.

As incubation progressed, βNTI values shifted progressively toward the neutral range (|βNTI| <2), indicating a transition from strong deterministic selection toward weaker deterministic or stochastic assembly dynamics. This temporal shift suggests that acute contaminant toxicity dominated early assembly processes, whereas later-stage community dynamics became increasingly influenced by secondary ecological processes such as resource competition, population recovery, and ecological drift.

Dose-dependent differences remained evident throughout the experiment. The high-dose treatment exhibited more persistent deviations in βNTI values, indicating sustained ecological filtering under elevated contaminant stress. Viral addition caused modest but significant increases in βNTI values under high-dose conditions at days 2 and 33 (*P* < 0.05), suggesting that viruses subtly influenced assembly dynamics under severe stress despite producing relatively minor compositional changes.

### Viral regulation suppresses respiration

**Fig 6:**
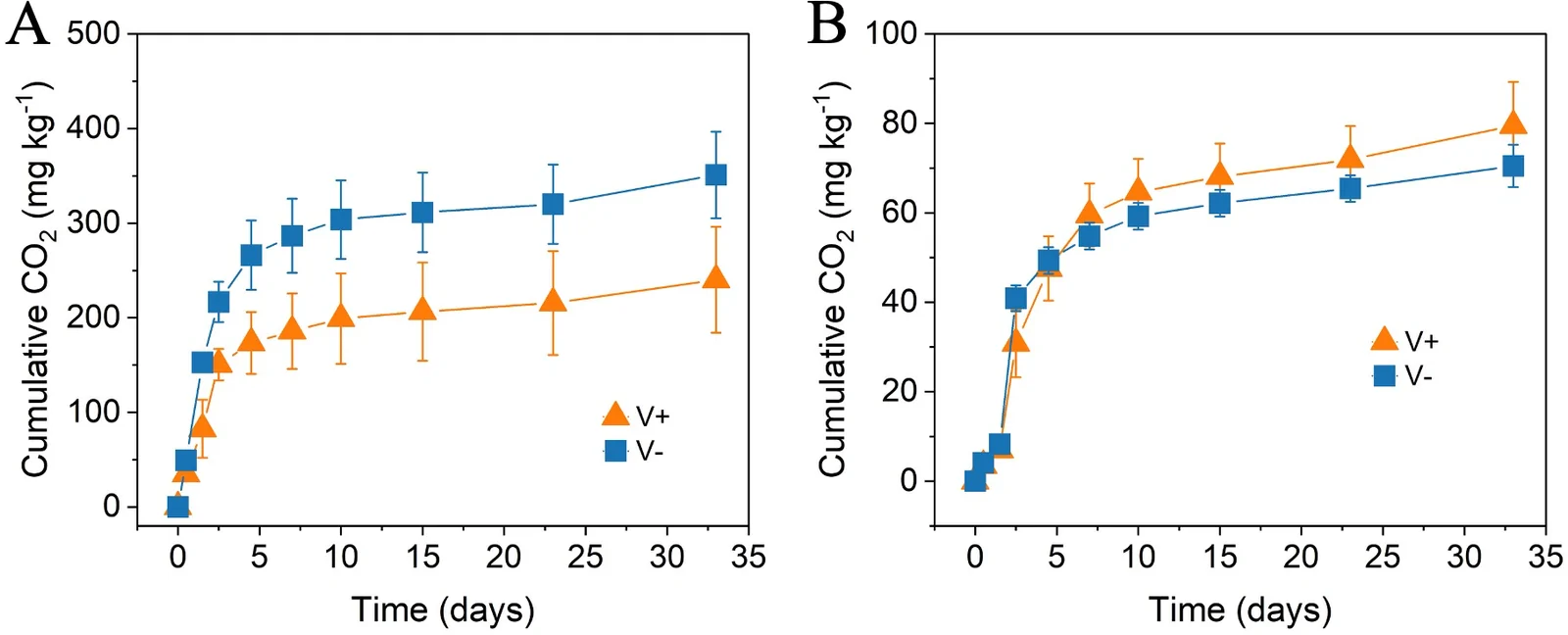
Microbial respiration (cumulative CO_2_-C evolution) at low (**A**) and high (**B**) doses over 33 days. V− and V+ denote virus-free and virus-present treatments.

In contrast to the strong dose-dependent effects on biodegradation and community assembly, viral addition primarily altered microbial respiratory activity rather than atrazine degradation (Fig. 6). Cumulative CO_2_ production increased rapidly during the first 7 days across all treatments, corresponding to the period of highest microbial metabolic activity.

Under the low-dose treatment, virus-present microcosms exhibited significantly lower cumulative respiration than virus-free controls (*P* = 0.008) (Fig. 6A). By day 33, cumulative CO_2_ production reached 351.1 ± 45.8 mg CO_2_/kg in virus-free microcosms but only 240.1 ± 55.8 mg CO_2_/kg in virus-present treatments. Despite this pronounced suppression of respiratory carbon flux, viral addition did not significantly alter atrazine degradation kinetics or major bacterial compositional metrics.

Under the high-dose treatment, overall respiration was strongly suppressed irrespective of viral presence, consistent with severe toxicity-driven metabolic inhibition (Fig. 6B). Nevertheless, the virus-present and virus-free treatments exhibited distinct temporal trajectories. During the first 5 days, virus-present microcosms showed lower cumulative respiration (47.5 ± 7.2 mg CO_2_/kg) than virus-free controls (49.3 ± 3.0 mg CO_2_/kg, *P* = 0.056). Notably, after day 5, virus-present treatments showed a modest increase in cumulative respiration (79.5 ± 9.8 mg CO_2_/kg) relative to virus-free controls (70.5 ± 4.7 mg CO_2_/kg); however, this difference was not statistically significant (*P* = 0.39, paired *t*-test).

## DISCUSSION

### Atrazine dose bifurcates microbial assembly between biodegradation and stress persistence

The clearest pattern emerging from this study is that atrazine dosage did not merely intensify microbial stress; rather, it redirected the ecological trajectory of the entire system. Low atrazine loading sustained communities capable of active biodegradation, whereas high loading shifted the microbiome toward stress persistence with sharply reduced catabolic performance. The transition between these two states behaved as an ecological threshold separating metabolically functional degrader-dominated assemblages from survival-oriented communities adapted primarily to withstand toxicity.

This dose-dependent behavior was reflected in both degradation kinetics and community succession. Under low-dose conditions, rapid enrichment of Pseudomonadota-associated taxa coincided with complete atrazine removal. Several enriched genera, including *Pseudomonas*, *Acinetobacter*, and *Aeromonas*, have previously been associated with atrazine mineralization and xenobiotic degradation pathways (21, 22). In contrast, the high-dose treatment initially favored Bacillota-dominated assemblages enriched in spore-forming and stress-tolerant taxa such as *Bacillus*, *Metabacillus*, and *Lysinibacillus*. These organisms are physiologically well suited to survive chemical stress yet are not typically regarded as dominant atrazine degraders. The resulting community therefore appeared metabolically adapted for stress tolerance rather than catabolic specialization.

Importantly, the divergence between these assembly trajectories cannot be interpreted simply as a compositional shift. Instead, the data suggest a reorganization of ecological strategy under increasing toxic stress. At moderate atrazine loading, the herbicide likely functioned simultaneously as a stressor and a selective substrate, enriching populations capable of coupling detoxification with growth. Once atrazine concentrations exceeded microbial physiological tolerance, however, selective pressure shifted toward persistence mechanisms that prioritized cellular survival over active contaminant transformation. The collapse of degradation at high dosage therefore appears to reflect a transition in ecological state rather than the mere loss of individual degrader taxa.

Microbial community assembly is a dynamic succession shaped by both deterministic and stochastic processes (23). The βNTI patterns reinforce this interpretation. Strongly negative βNTI values immediately following atrazine exposure indicate intense deterministic filtering during the early assembly stage, consistent with acute toxicity rapidly excluding sensitive taxa. As incubation progressed, βNTI values approached the stochastic range, implying that the influence of direct toxic filtering weakened over time as surviving populations stabilized and secondary ecological processes became increasingly important. In this context, early community assembly was governed primarily by physiological tolerance, whereas later succession reflected competition among the taxa that successfully passed the initial environmental filter. However, we acknowledge a limitation in the temporal resolution of our assembly analysis. The critical first 48 h—when acute toxic stress likely exerts the strongest deterministic filtering—were not captured in our βNTI analysis. Consequently, the inferred trajectory from deterministic to stochastic assembly should be interpreted as a coarse temporal approximation.

This framework helps reconcile why contaminant persistence often changes abruptly rather than gradually across concentration gradients in agricultural soils (24, 25). Once toxic loading surpasses the functional tolerance of degrader populations, the system may reorganize toward stress survival with limited biodegradation potential (10, 26). Such threshold responses likely represent an underappreciated feature of contaminant ecology and may explain the high variability in biodegradation outcomes reported across polluted environments.

### Specialized biodegradation and community-scale metabolism exhibit fundamentally different sensitivities

One of the most striking findings of this study is that atrazine biodegradation and microbial respiration responded differently to both contaminant stress and viral modulation. Although these processes are frequently treated as linked indicators of microbial functioning, the present results indicate that they represent ecologically distinct functions operating at different organizational levels within the microbiome. “Functional decoupling” is defined here as the independent response of two microbial ecosystem functions—specialized atrazine degradation and community-scale respiration—to the same environmental perturbation. This decoupling was clearly demonstrated under the low-dose regime, where viral addition significantly suppressed respiration while atrazine degradation persisted, implying that these two functions are regulated by distinct ecological and physiological controls.

Atrazine degradation constitutes a relatively specialized metabolic capability restricted to subsets of the community possessing appropriate catabolic pathways (27). As long as these key functional populations remain active, biodegradation can persist even when broader microbial diversity and total metabolic activity are substantially reduced (28). Respiration, by contrast, reflects aggregate community-scale carbon flux arising from maintenance metabolism, growth-associated energy demand, stress responses, and turnover across the entire microbial assemblage. It therefore integrates the physiological state of the broader community rather than the persistence of specific catabolic guilds.

This distinction provides a mechanistic explanation for the functional decoupling observed here. Under low-dose conditions, viral addition substantially reduced microbial respiration without altering atrazine degradation kinetics. The degrader populations responsible for atrazine transformation therefore remained sufficiently functional despite strong suppression of overall respiratory activity. In other words, the metabolic threshold required to sustain specialized catabolism remained intact even as whole-community carbon processing declined.

The ecological implications of this distinction are important. In contaminated systems, broad metabolic indicators such as respiration or biomass may not reliably predict contaminant degradation capacity (11). Communities can retain specialized biodegradation functions despite pronounced reductions in total metabolic activity, provided that the responsible functional guilds remain ecologically stable (10). Conversely, communities may remain metabolically active while losing effective degradation capacity if key catabolic taxa are selectively eliminated.

The results of microbial community diversity are also consistent with this interpretation. Richness declined more strongly than Shannon diversity under elevated atrazine stress, indicating a preferential loss of low-abundance taxa. Such patterns suggest that contaminant exposure initially compresses the breadth of ecological strategies within the community while allowing a limited number of dominant stress-adapted populations to persist. The resulting communities may therefore retain specific specialized functions while simultaneously losing broader metabolic versatility and ecological redundancy. The microbial responses to contaminants are inherently multidimensional (8). Different ecosystem functions occupy different positions along the spectrum of physiological sensitivity, ecological redundancy, and taxonomic specialization. Treating microbial functioning as a single unified property risks obscuring these important distinctions.

### Viral regulation was expressed primarily through metabolic suppression rather than taxonomic restructuring

Compared with the pronounced effects of atrazine dosage, viral addition exerted relatively subtle effects on bacterial composition yet disproportionately strong effects on microbial respiration. This asymmetry is central to interpreting the ecological role of viruses under contaminant stress. The absence of major viral effects on community composition indicates that viruses did not substantially reorganize dominant bacterial assemblages during the incubation period. Nonetheless, respiration declined markedly in virus-amended low-dose microcosms, demonstrating that viral influence emerged primarily through altered metabolic activity rather than compositional turnover. Viral regulation therefore became detectable at the level of carbon flux long before it became apparent through conventional taxonomic metrics (29).

Several mechanisms could contribute to this pattern. Viral infection can reduce host metabolic efficiency, suppress population growth, redirect cellular resources toward stress maintenance, or increase microbial turnover without necessarily altering dominant taxonomic structure (30, 31). Lytic infection may reduce host metabolic efficiency by redirecting cellular resources toward viral progeny production, while rapid population turnover may sustain taxonomic structure at the cost of increased maintenance respiration. Alternatively, lysogenic viruses may impose a cryptic metabolic burden on hosts, particularly under contaminant stress, without triggering lytic cycles. Finally, virus-mediated release of dissolved organic matter could indirectly alter the metabolic state of non-infected populations. Importantly, the current data do not directly resolve which of these mechanisms predominated here. The study did not quantify infection rates, lysogeny dynamics, or viral gene expression, and therefore strong mechanistic claims regarding auxiliary metabolic genes or viral life-history transitions would not be sufficiently supported. The present results instead point more conservatively toward a metabolic effect operating at the scale of whole-community activity.

This distinction may be especially relevant in contaminant-stressed soils. Toxic environments already impose strong energetic constraints on microbial populations, potentially narrowing the physiological margin between growth and maintenance metabolism (8). Under such conditions, relatively modest viral effects could disproportionately alter respiratory carbon loss while leaving specialized catabolic pathways operational (32). The respiratory sensitivity observed here may therefore reflect the vulnerability of broad community metabolism under combined chemical and viral stress. Critically, rather than acting as dominant drivers of community restructuring, viruses in this system appeared to function as modulators of metabolic intensity (16). This interpretation aligns more closely with the observed data than models emphasizing large-scale viral control of biodegradation or bacterial composition.

### Ecological implications for contaminant fate and soil carbon cycling

The ecological significance of these findings extends beyond atrazine itself. Most frameworks assume microbial functions respond coherently to disturbance, but our study shows they can diverge substantially in sensitivity. Specialized biodegradation may persist across broader disturbance ranges than general metabolism, provided degraders survive initial filtering. Thus, reduced respiration or diversity does not necessarily signal loss of degradation capacity. However, once contaminant loading exceeds tolerance thresholds, systems may rapidly reorganize toward stress persistence with limited biodegradation. Viral suppression of respiration also indicates that viruses influence carbon cycling independently of contaminant removal. More broadly, specialized catabolism, general metabolism, stress physiology, and assembly dynamics each respond to distinct constraints. Understanding contaminant ecology therefore requires a process-based framework integrating ecological filtering, functional specialization, and metabolic regulation, where communities reorganize across multiple functional dimensions rather than being simply “suppressed” or “resistant.”

The use of sterilized sand microcosms represents a simplification of natural soil ecosystems here. In agricultural soils, atrazine bioavailability is modulated by organic matter sorption, clay mineral interactions, and complex microbial food webs, all of which were excluded from our system. Consequently, the degradation rates and community responses observed here may not directly translate to field conditions. Nevertheless, this reductionist design was intentionally chosen to test causal ecological mechanisms under controlled conditions, providing a clear baseline for future studies that incorporate increasing environmental complexity.

### Conclusion

This study reveals a functional decoupling between atrazine biodegradation and microbial respiration under contaminant stress and viral modulation. Atrazine dose established the primary ecological responses. Low-dose exposure selected degrader-dominated communities capable of complete biodegradation, whereas high-dose exposure redirected assembly toward stress-tolerant populations and suppressed catabolic performance. Thus, atrazine persistence was governed not simply by microbial presence, but by whether contaminant loading permitted communities to organize around active degradation rather than survival. Viral addition constituted a secondary level of regulation, distinct from, and subordinate to, the primary effects of atrazine dose. Although viruses did not measurably alter atrazine degradation kinetics or dominant bacterial community structure, they significantly reduced respiratory carbon loss under the low-dose regime. This contrast indicates that specialized biodegradation capacity and community-scale metabolic flux differ in their sensitivity to viral regulation. Atrazine degradation remained resilient as long as key degrader assemblages persisted, whereas respiration responded strongly to viral modulation of microbial activity. These findings caution against treating microbial ecosystem functions as uniformly responsive to contaminant stress. Predicting pesticide fate and soil carbon consequences requires distinguishing specialized catabolic processes from broader metabolic fluxes, and integrating microbial assembly, functional thresholds, and viral regulation into a unified framework.

## Data Availability

Raw sequencing data have been deposited in the NCBI Sequence Read Archive under BioProject accession number PRJNA1468458.
